# Arterial occlusive events in chronic myeloid leukemia patients treated with ponatinib in the real‐life practice are predicted by the Systematic Coronary Risk Evaluation (SCORE) chart

**DOI:** 10.1002/hon.2606

**Published:** 2019-04-17

**Authors:** Giovanni Caocci, Olga Mulas, Elisabetta Abruzzese, Luigiana Luciano, Alessandra Iurlo, Immacolata Attolico, Fausto Castagnetti, Sara Galimberti, Nicola Sgherza, Massimiliano Bonifacio, Mario Annunziata, Antonella Gozzini, Ester Maria Orlandi, Fabio Stagno, Gianni Binotto, Patrizia Pregno, Claudio Fozza, Malgorzata Monika Trawinska, Fiorenza De Gregorio, Daniele Cattaneo, Francesco Albano, Gabriele Gugliotta, Claudia Baratè, Luigi Scaffidi, Chiara Elena, Francesca Pirillo, Emilia Scalzulli, Giorgio La Nasa, Robin Foà, Massimo Breccia

**Affiliations:** ^1^ Hematology Unit, Businco Hospital, Department of Medical Sciences and Public Health University of Cagliari Cagliari Italy; ^2^ Hematology Unit Sant'Eugenio Hospital Tor Vergata University Rome Italy; ^3^ Hematology Unit “Federico II” University of Naples Naples Italy; ^4^ Hematology Unit Fondazione IRCCS Ca' Granda Ospedale Maggiore Policlinico Milan Italy; ^5^ Department of Emergency and Organ Transplantation—Hematology Section University of Bari Bari Italy; ^6^ Department of Experimental, Diagnostic and Specialty Medicine, S. Orsola‐Malpighi Hospital University of Bologna Bologna Italy; ^7^ Department of Clinical and Experimental Medicine, Section of Hematology University of Pisa Pisa Italy; ^8^ Hematology and Transplant Center Casa Sollievo della Sofferenza Hospital San Giovanni Rotondo Italy; ^9^ Department of Medicine, Section of Hematology University of Verona Verona Italy; ^10^ Hematology Unit Cardarelli Hospital Naples Italy; ^11^ Hematology Unit, AOU Careggi University of Florence Florence Italy; ^12^ Division of Hematology “Fondazione IRCCS Policlinico S. Matteo” Pavia Italy; ^13^ Hematology Unit AOU Policlinico—V. Emanuele, Rodolico Hospital Catania Italy; ^14^ Hematology Unit University of Padova Padua Italy; ^15^ Hematology Unit Azienda Ospedaliero‐Universitaria Città della Salute e della Scienza Torino Italy; ^16^ Department of Medical, Surgical and Experimental Sciences University of Sassari Sassari Italy; ^17^ Hematology, Department of Cellular Biotechnologies and Hematology Sapienza University Rome Italy

**Keywords:** arterial occlusive event, chronic myeloid leukemia, ponatinib, prophylaxis

## Abstract

Arterial occlusive events (AOEs) represent emerging complications in chronic myeloid leukemia (CML) patients treated with ponatinib. We identified 85 consecutive CML adult patients who were treated with ponatinib in 17 Italian centers. Patients were stratified according to the Systematic Coronary Risk Evaluation (SCORE) assessment, based on sex, age, smoking habits, systolic blood pressure, and total cholesterol levels.

The 60‐month cumulative incidence rate of AOEs excluding hypertension was 25.7%. Hypertension was reported in 14.1% of patients. The median time of exposure to ponatinib was 28 months (range, 3‐69 months). Patients with a high to very high SCORE risk showed a significantly higher incidence rate of AOEs (74.3% vs 15.2%, *P* < 0.001). Patients aged ≥60 years showed a significantly higher incidence rate of AOEs (51.5% vs 16.9%, *P* = 0.008). In multivariate analysis, no association was found between AOEs and positive history of CV disease, age, dose of ponatinib, previous exposure to nilotinib, and comorbidities. Only the SCORE risk was confirmed as a significant predictive factor (*P* = 0.01; HR = 10.9; 95% C.I. = 1.7‐67.8). Patients aged ≥60 years who were treated with aspirin had a lower incidence rate of AOEs (33.3% vs 61.8%). Among the 14 reported AOEs, 78.6% of them showed grade 3 to 4 toxicity. This real‐life study confirmed the increased incidence of AOEs in CML patients treated with ponatinib, with high to very high SCORE risk. We suggest that patients aged ≥60 years who were treated with ponatinib should undergo prophylaxis with 100 mg/day of aspirin. Our findings emphasize personalized prevention strategies based on CV risk factors.

## 1 INTRODUCTION

Ponatinib is a third‐generation tyrosine kinase inhibitor (TKI), active against native and mutated BCR‐ABL1, indicated for the treatment of chronic myeloid leukemia (CML) patients in every phase of the disease, resistant and/or intolerant to dasatinib and nilotinib, with or without T315I mutation.^1^ Unfortunately, ponatinib treatment may induce cardiovascular adverse events (CVAEs) and, in particular, arterial occlusive events (AOEs). In the 5‐year follow‐up of the multinational phase II Ph + ALL and CML Evaluation (PACE) trial, the cumulative incidence rate of AOEs was 31% in the chronic‐phase CML population; the cumulative incidence rate of AOEs continued to increase over time, but the exposure‐adjusted incidence of newly occurring AOEs remained relatively constant throughout the study, due to the reduction of the dose since October 2013.^2^ The incidence rate of AOEs following ponatinib treatment in real life was reported only in a few small patient cohorts followed up for short periods, showing variable outcomes.^3^, ^4^, ^5^, ^6^ Risk factors associated with the development of AOEs have been identified in basal CV risk factors and/or may include the following: a history of previous ischemic disease,^2^ dose intensity and age at starting ponatinib,^7^ male sex, previous history of AOEs, and previous exposure to nilotinib.^5^ The usefulness of the Systematic Coronary Risk Evaluation (SCORE) risk assessment at disease baseline, a 10‐year risk estimation of fatal CV disease based on sex, age, smoking, systolic pressure, and total cholesterol level, to identify patients with increased risk of occurrence of AOEs during ponatinib treatment has been suggested.^8^, ^9^

Given the growing interest on the occurrence of AOEs in CML patients as off‐target effects in the long term and the emergence of a new interdisciplinary specialty of “cardio‐oncology” focusing on the proper stratification of CV risk in CML patients and management of complications, ad hoc expert opinions in this field have been published.^9^, ^10^ Overall, the recommendations agree on the importance of the CV risk stratification at baseline and suggest that certain TKIs should be used with caution in patients with preexisting CV risk factors, highlighting the need for a careful monitoring prior to the administration of the drug and during treatment.

Moreover, in CML patients treated with ponatinib, a preventing strategy with primary prophylaxis based on aspirin is still under discussion: the use of an antiplatelet agent in CML is not supported by literature data or by guidelines and is so far based on medical decision on an individual basis.

We therefore reported a large real‐life cohort of Italian CML patients treated with ponatinib outside clinical trials. The primary endpoint was to establish the incidence of AOEs and the association with the SCORE assessment and baseline CV risk factors. Secondary endpoints were to evaluate the role of primary prophylaxis in preventing AOEs and to report the management of AOE complications in the clinical practice.

## 2 METHODS

We identified 85 consecutive nonselected adult CML patients who were treated with ponatinib outside clinical trials between January 2012 and December 2017 in 17 Italian centers. Information on CV risk factors before starting ponatinib was retrospectively collected from the review of medical charts. To estimate the SCORE risk, all patients were evaluated at diagnosis for age, gender, tobacco use, systolic pressure, and total cholesterol serum level; patients were stratified into low to moderate (SCORE ≤ 5%) or high to very high (SCORE risk >5%) CV risk. Additional risk factors were as follows: presence of diabetes, body mass index >24.5 kg/m^2^, mild or severe renal insufficiency, and dyslipidemia. Patients were also evaluated for comorbidities and a positive anamnesis of CV diseases, including myocardial infarction, heart failure, cardiomyopathy, angina, stroke, arterial hypertension, valvular heart disease, heart arrhythmia, aortic aneurysms, peripheral artery disease, ischemic cerebrovascular events, venous thrombosis, and thromboembolic disease. The presence of antithrombotic prophylaxis before starting CML treatment was also recorded and, according to the same indications as the general population, defined as primary (based on the presence of risk factors) prophylaxis, mainly represented by 100 mg/day of aspirin, or secondary prophylaxis (positive history of atherothrombotic events), represented by aspirin, other antiplatelet agents such as ticlopidine or clopidogrel, or anticoagulant therapy. We evaluated the cumulative incidence rate of AOEs (CV excluding hypertension, cerebrovascular, and peripheral vascular) after initiating the treatment with ponatinib and their management by the hematologists and cardiologists.

CML patients' response to TKI was evaluated according to the European Leukemia Net recommendations.^11^ Molecular response was estimated by the presence of detectable BCR‐ABL transcripts using quantitative reverse transcription‐polymerase chain reaction with a sensitivity of 3 logs (MR^3^) or deeper (MR^4^).^12^

The probability of the cumulative incidence rate of AOEs was estimated after administering ponatinib. The cumulative incidence rate of MR^4^ was evaluated from the start of ponatinib treatment. The log‐rank test was used to compare two or more groups of stratified patients. We evaluated the impact of the following variables on the incidence of AOEs: positive anamnesis for CV disease, age ≥ 60 years, ponatinib dose, previous exposure to nilotinib, comorbidities, primary prophylaxis, and SCORE risk. Multivariate analyses were performed using the Cox proportional hazards regression model. A *P*‐value <0.05 was considered statistically significant. Data analysis was performed using a standard statistical package (SPSS for Macintosh, Version 21, Chicago, IL).

## 3 RESULTS

### 3.1 Patients' characteristics, arterial occlusive events, and clinical outcomes

A total of 85 consecutive chronic CML patients were retrospectively identified. The patients' characteristics are shown in Table 1. The median age at diagnosis was 53 years (range, 23‐81 years). The Sokal score was intermediate to high in 58.9% of patients. The median interval since CML diagnosis was 6.3 years (range, 0.8‐25.9 years). The reasons for treatment with ponatinib included the following: inefficacy of previous TKI in 81.1% and intolerance in 18.9% of patients. At ponatinib starting, 21 patients (24.7%) harbored a T415I mutation. In the majority of cases, ponatinib was scheduled as a third‐line or subsequent lines of treatment. Ponatinib was administered at the following doses: 45 mg/day in 40% of patients, 30 mg/day in 42.3% of patients, and 15 mg/day in 17.7% of patients, respectively.

**Table 1:** Characteristics of 85 CML patients treated with ponatinib

Sex	N°	%
Male	47	(55)
Female	38	(45)
*Age at diagnosis, median years (range)*	53	(23‐81)
*Median follow‐up, median years (range)*	6.3	(0.8‐25.9)
*Leukocyte ×10* ^*3*^ */uL, mean value (range)*	104	(7‐500)
*Hemoglobin g/dl, mean value (range)*	12.7	(5.5‐16.7)
*Platelet ×10* ^*3*^ */uL, mean value (range)*	400	(45‐1667)
*BCR‐ABL transcript type*		
p210	83	(97.6)
p190	2	(2.4)
p230	0	(0)
*Splenomegaly*	48	(56.5)
*Sokal score*		
Low	35	(41.1)
Intermediate	36	(42.4)
High	14	(16.5)
*Line of treatment*		
Second line	23	(27)
Third line	37	(43.5)
Fourth line	25	(29.5)
*Ponatinib dose*		
45 mg/day	34	(40)
30 mg/day	36	(42.3)
15 mg/day	15	(17.7)
*Reason of switch*		
Inefficacy	69	(81.1)
Intolerance	16	(18.9)
Duration of ponatinib *median months (range)*	28	(3‐69)

**Figure 1:**
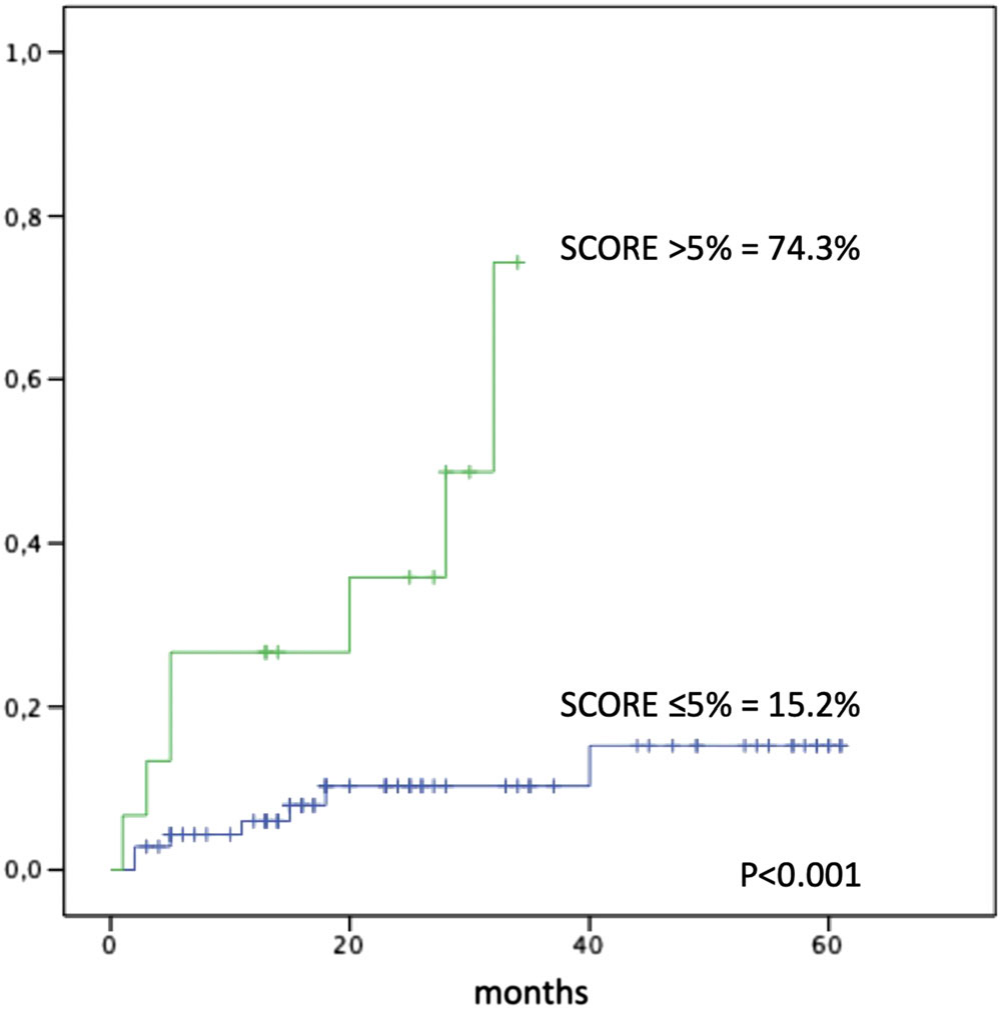
Cumulative incidence of arterial occlusive events in 85 patients treated with ponatinib, according to the SCORE risk, based on sex, age, smoking habits, systolic blood pressure, and total cholesterol levels

The median time of exposure to ponatinib was 28 months (range, 3‐69 months). At the time of AOE occurrence, 14.3% of patients received 45 mg/day of ponatinib, 64.3% of patients received 30 mg/day of ponatinib, and 21.4% of patients received 15 mg/day of ponatinib, respectively. The median time between the start of ponatinib treatment and the occurrence of AOEs was 16 months (range, 1‐61 months). A positive history for CV diseases was reported in 32 patients (37.7%). The 60‐month cumulative incidence rate of AOEs was 25.7 ± 7%. Hypertension was reported in 14.1% of patients. The majority of the patients (82.3%) were classified at low to intermediate risk (SCORE risk ≤5%) and 17.7% of patients at high to very high risk (SCORE risk >5%) according to the SCORE risk chart evaluation. Patients with a high to very high SCORE risk showed a significantly higher incidence of AOEs (74.3 ± 19.7% vs 15.2 ± 6.2%, *P* < 0.001) (Figure 1). Patients aged ≥60 years showed a significantly higher incidence of AOEs (51.5 ± 16.6% vs 16.92 ± 6.9, *P* = 0.008).

In multivariate analysis, no significant association was found between AOEs and positive history of CV disease, age, dose of ponatinib, previous exposure to nilotinib, and comorbidities. Only the SCORE risk was confirmed to have a significant association (*P* = 0.01; hazard ratio = 10.9; 95% confidence interval = 1.7‐67.8) with the incidence of AOEs. Finally, the 5‐year cumulative incidence rate of MR^4^ following ponatinib treatment was 40.6 ± 10.7%, and it was not influenced significantly by AOE occurrence.

### 3.2 Role of primary prophylaxis

Overall, 19 patients were treated with 100 mg/day of aspirin as a primary prophylaxis before starting the administration of ponatinib.

In these patients, the 25‐year overall survival rate was 94.1 ± 5.7% in comparison with 76.4 ± 10.8% in 66 patients who did not receive primary prophylaxis (*P* = NS).

No significant association was found between AOE occurrence and primary prophylaxis. Nevertheless, in patients aged ≥60 years treated with aspirin, we found a lower, although not statistically significant, incidence rate of AOEs (33.3 ± 27.2% vs 58.6 ± 19.2%, *P* = NS). In six patients aged ≥60 years taking aspirin, only one AOE was registered, in comparison with six AOEs in 15 older patients not taking aspirin.

### 3.3 Management of CV events

**Figure 2:**
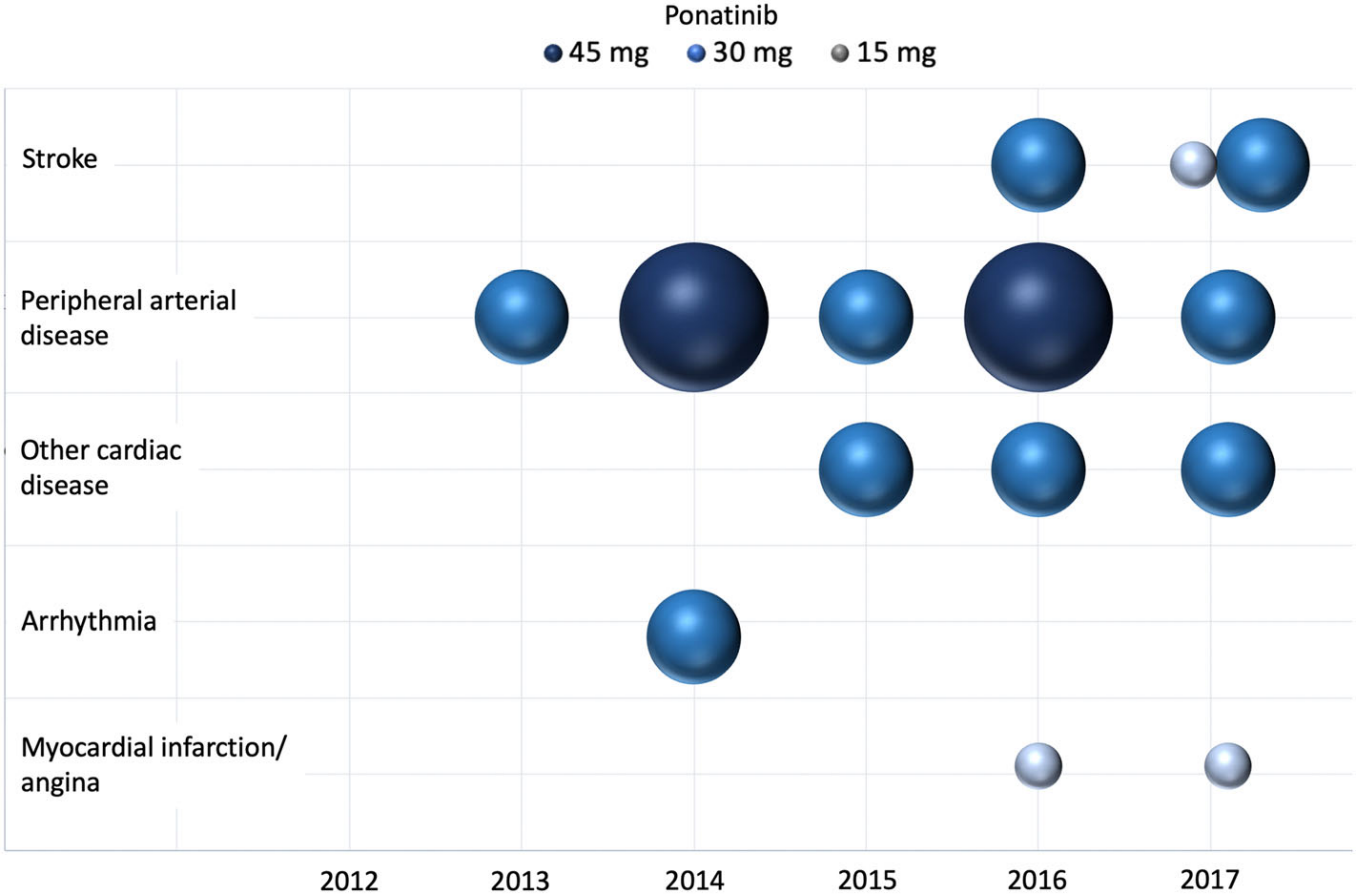
Bubble chart displaying 14 occlusive events that occurred from 2012 to 2017 in 85 patients treated with different doses of ponatinib. The diameter of the area of the bubble represent the dose of ponatinib (15, 30, or 45 mg)

Overall, 26 CVAEs in 80 patients were recorded: 14 AOEs and 12 hypertension cases (Figure 2, Table 2); 13 CVAEs were graded as 3 to 4 according to the common toxicity criteria.^13^ At the time of CVAEs, five patients were administered with 15 mg/day (19.2% of CVAEs), eight with 30 mg/day (30.8% of CVAEs), and 13 patients with 45 mg/day (50% of CVAEs) of ponatinib. Dosing during AOEs is reported in Figure 2. A higher drug dose was not associated to AOEs. Following the CVAEs, 11 patients undergoing ponatinib treatment did not require dose modification, four patients reduced the dose, and 11 patients discontinued the treatment. Majority of the patients were required to undergo additional diagnostic tests such as ECG/cardiac ultrasound, peripheral vascular Doppler ultrasound, and cranial CT; two patients underwent coronarography, and three patients required invasive procedures such as percutaneous transluminal angioplasty and coronary stent application. Additional medical therapy was introduced in the majority of cases.

**Table 2:** Cardiovascular profile of 85 patients and management of 26 cardiovascular adverse events

	N°	(%)			N°	(%)
*CV risk factors*				*TKI combinations in CVAEs*		
Hypertension	20	(23.5)		Ima‐Pona	1	(1.2)
Dyslipidemia	24	(28.2)		Dasa‐Pona	3	(3.5)
Obesity (BMI >24.5)	49	(57.6)		Nilo‐Pona	1	(1.2)
Severe renal insufficiency	1	(1.2)		Ima‐Dasa‐Pona	5	(5.8)
Diabetes	12	(14.1)		Ima‐Nilo‐Pona	5	(5.8)
SCORE^a^ risk ≤5% (low to int.)	70	(82.3)		Ima‐Dasa‐Nilo‐Pona	5	(5.8)
SCORE risk >5% (high to very high)	15	(17.7)		Ima‐Nilo‐Dasa‐Pona	5	(5.8)
				Ima‐Bosu‐Ima‐Pona	1	(1.2)
*CVD before ponatinib treatment*						
Myocardial infarction/angina	4	(4.7)		*Toxicity grading CVAEs*		
Arrhythmia	3	(3.5)		Grade 1‐2	13	(15.3)
Other cardiac diseases^b^	5	(5.8)		Grade 3‐4	13	(15.3)
Peripheral arterial disease	0	(0)		Grade 5	0	(0)
Stroke	0	(0)				
Hypertension	20	(23.5)		*Ponatinib dose modification*		
Peripheral venous disease	0	(0)		Unchanged	11	(13)
Primary prophylaxis^c^	17	(20)		Reduced	4	(4.7)
Secondary prophylaxis^d^	6	(7)		Interrupted	11	(13)
						
*CVAEs following ponatinib*				*Additional tests requested*		
Number of CVAEs	26	(30.5)		Coronarography	2	(2.3)
Number of AOEs	14	(16.5)		ECG/cardiac ultrasound	12	(14.1)
Myocardial infarction/angina	2	(2.3)		Cardiac angio‐MR/TAC	4	(4.7)
Arrhythmia	1	(1.2)		Peripheral vascular Doppler ultrasound	2	(2.3)
Other cardiac diseases^e^	3	(3.5)		Nothing	7	(8.2)
Peripheral arterial disease^f^	5	(5.8)				
Stroke	3	(3.5)		*Therapies introduced*		
Hypertension	12	(14.1)		Coronary stents	2	(2.3)
				PTA^f^ peripheral artery	1	(1.2)
*Ponatinib dose at CVAEs*				Antiplatelet	8	(9.4)
45 mg/day	13	(15.3)		Anticoagulant	1	(1.2)
30 mg/day	8	(9.4)		Antiarrhythmic	1	(1.2)
15 mg/day	5	(5.8)		Other drugs®	14	(16.5)
				No further action	2	(2.3)
*Line of treatment*						
Second line	5	(5.8)		*Total CV‐related deaths*	0	(0)
Third line	10	(11.8)				
Fourth line	11	(13)					

Abbreviations: AOEs, arterial occlusive events; CVAES, cardiovascular adverse events; CVD, cardiovascular disease.

a Based on sex, age, smoking, systolic pressure, and cholesterol level.

b Valvulopathy and dilated cardiomyopathy.

c Aspirin.

d Aspirin, clopidogrel, and ticlopidine.

e QT elongation and cardiac failure.

f PAOD (peripheral arterial occlusive disease) and atheromatous carotid disease.

PTA (percutaneous transluminal angioplasty).

Diuretics, calcium channel blockers, ACE inhibitors, and beta blockers.

## 4 DISCUSSION

AOEs represent off‐target relevant complications of TKIs, particularly second‐generation and third‐generation TKIs.^14^ Nilotinib and ponatinib interact with a considerable number of clinically relevant vascular targets implicated in endothelial cell survival and angiogenesis.^15^

Ponatinib was temporarily suspended in 2013 as its administration resulted in the occurrence of CV thrombotic events, and since then, several publications have provided general management recommendations for patients eligible to receive ponatinib.^9^, ^10^ The cumulative incidence rate of AOEs reported by the 5‐year follow‐up of PACE trial was 31% (serious AOEs, 26%) in the chronic‐phase CML population; in this report, the higher cumulative incidence rate correlated with the longer duration of treatment with ponatinib.^2^ Independent variables significantly associated with AOEs were basal CV risk factors and a history of ischemic disease; notably, even after a suggested preemptive dose reduction in October 2013, the percentage of patients who had experienced the first occurrence of AOE was similar in the cohort of patients who underwent the dose reduction and those who maintained treatment at the same dose.^2^ Company‐sponsored CML randomized clinical trials investigating ponatinib efficacy and safety might have important selection biases because they are not designed to register baseline traditional CV risk factors and because of their correlation with AOEs. Nevertheless, a systematic review and multivariate analysis from a pooled population including three clinical trials showed that dose intensity, history of ischemic disease, and age were the strongest independent predictors of increased risk of AOE; this model predicted an approximately 33% reduction in the risk of AOE for each 15 mg/day decrease in average ponatinib dose intensity.^7^

Recently published data originating from real life, based on a small population cohort study, reported the absence or very low incidence of AOEs.^3^, ^4^ The authors enumerated the following possible reasons of these unexpected data: the limited follow‐up, a better selection of patients with few concomitant comorbidities, a lack of preexisting CV risk factors at baseline, a low starting dose of the drug in 40% of patients, the number of TKI lines of treatment,^3^ and significantly younger population in comparison with the PACE trial.^4^ Other recent studies from real life reported a variable incidence rate of CVAEs from 23% to 56%, suggesting that male sex, previous history of AOEs, and previous exposure to nilotinib are considered risk factors.^5^, ^6^

Overall, these observations suggested the need to customize treatment approaches, considering the appropriate TKI selection according to baseline CV risk factors.

We evaluated a retrospective large cohort of 85 consecutive CML patients managed in Italy and treated with ponatinib in subsequent lines of treatment and found a 5‐year AOE (excluding hypertension) cumulative incidence rate of 25.7%, similar to the PACE trial (31%), which included hypertension as one of the AOEs. Our study showed that patients aged ≥60 years represented a risk factor in a univariate analysis. Age was found to be associated to AOEs also in the published multivariate analysis including 671 ponatinib‐treated patients.^7^ Moreover, we confirmed for the first time that the SCORE risk chart, based on sex, age, smoking, systolic pressure, and total cholesterol level, in the setting of ponatinib‐treated patients has an independent predictive value; indeed, patients with a high to very high risk score reported a significantly higher incidence of AOEs (74.3% vs 15.2%, *P* < 0.001), and this risk factor remained significant also in multivariate analysis (Figure 1). Overall, personalized strategies to minimize the risk of AOEs should be thoroughly screened; this could be particularly important for the elderly patients with multiple comorbidities. No significant differences in AOE incidence were found according to the different TKI combinations registered in the subsequent lines of treatment or the different doses of ponatinib (15 mg/day, 30 mg/day, 45 mg/day) received by the patients (Table 2). Overall, ponatinib efficacy was confirmed in this cohort of heavily pretreated patients, reaching a 60‐month cumulative incidence rate of 40.6% in MR^4^, and this outcome was not influenced by the occurrence of AOEs.

Among the 14 reported AOEs, 78.6% of them showed a grade of 3 to 4 toxicity. The frequencies of CV, cerebrovascular, and peripheral vascular disease (PAOD or atheromatous carotid disease) were 14.3%, 21.4%, and 35.7%, respectively. The dose of ponatinib was reduced in 7.1% of patients, and ponatinib administration was discontinued in 78.6% of patients. Additional diagnostic tests were requested in most cases, such as coronarography, ECG/cardiac ultrasound, cardiac angio‐MR/CT, and peripheral vascular Doppler ultrasound. Additional drugs were administered in the majority of patients, and invasive procedures (coronary stents and percutaneous transluminal angioplasty) were performed in three patients (Table 2). Ideally, management and treatment of these patients should be carried out in close collaboration with cardiologists, angiologists, and vascular surgeons.

Notably, primary prophylaxis with 100 mg/day of aspirin may represent an option for this category of patients, aiming at reducing the incidence of atherothrombotic events.

Although the role of aspirin is still a matter of debate and no data have thus far been published, we observed a lower, although not statistically significant, incidence rate of AOEs (16.9% vs 51.5%) in patients aged ≥60 years who were treated with 100 mg/day of aspirin.

Future prospective studies are needed to further corroborate our preliminary findings.

Given the long‐term (often lifelong) TKI treatment required by the majority of CML patients, who nowadays can expect a survival similar to that of their peers from the general population,^16^ a personalized treatment approach, based not only on disease‐free survival but also on safety and quality of life, is needed.^17^, ^18^, ^19^ This aim requires the availability of a cardio‐oncology facility, with cardio‐oncology a discipline based on the collaboration between cardiologists, hematologists, and other medical specialists with the aim of preventing, monitoring, diagnosing, and treating AOEs before, during, and after treatment. Several steps to prevent AOEs in CML patients treated with TKIs have been suggested.^20^ Besides the management of modifiable CV risk factors, primary prophylaxis with aspirin has been proposed for “selected” patients.

In conclusion, this study confirms the increased risk of AOEs in CML patients treated with ponatinib in real life, harboring a high to very high SCORE risk. Our findings emphasize the need of personalized prevention strategies based on CV risk factors in close collaboration with cardio‐oncologists, angiologists, and vascular surgeons. We suggest that patients aged ≥60 years treated with ponatinib should undergo a prophylaxis with 100 mg/day of aspirin. Data on the efficacy of the primary prophylaxis need to be confirmed in larger cohorts of patients and in prospective randomized trials.

## DECLARATIONS

Ethics approval and consent to participate: Data on patients were retrospectively collected in accordance with the 1975 guidelines of the Declaration of Helsinki.

Competing interests: The other authors have no conflicts of interest to disclose.

Funding: None.

Authors' contributions:

GC and MB conceptualized and designed the study; GC, OM, EA, LL, AI, IA, FC, SG, NS, MB, MA, AG, AMO, FS, GB, PP, CF, MMT, FDG, DC, FA, GG, CB, LS, CE, FP, ES, GLN, RF, and MB collected and assembled the data; GC and OM performed the statistical analysis; GC and MB wrote the manuscript; and GC, OM, EA, LL, AI, IA, FC, SG, NS, MB, MA, AG, AMO, FS, GB, PP, CF, MMT, FDG, DC, FA, GG, CB, LS, CE, FP, ES, GLN, RF, and MB were responsible for the final approval of the manuscript.
